# The role of endoplasmic reticulum stress-mediated autophagy in cadmium-induced liver injury in rats

**DOI:** 10.3389/fvets.2026.1868085

**Published:** 2026-07-03

**Authors:** Chengxiang Guo, Hao Ling, Junbing Mao, Jiale Dong, Cai Zhang, Yue Wang, Shuai Wang, Shuai Guo, Jicang Wang

**Affiliations:** 1 College of Animal Science and Technology, Henan University of Science and Technology, Luoyang, China; 2 Henan Key Laboratory of Environmental and Animal Product Safety, Henan University of Science and Technology, Luoyang, China

**Keywords:** apoptosis, autophagy, cadmium, endoplasmic reticulum stress, liver injury

## Abstract

Cadmium (Cd) is a widespread toxicant with high bioaccumulation potential. This study explores the interplay of endoplasmic reticulum stress (ERS), autophagy, and apoptosis in Cd-induced hepatotoxicity, focusing on whether ERS-driven autophagy protects against liver injury. Male SD rats (4 weeks old, *n* = 24) were acclimatized for 7 days and randomized into four groups receiving CdCl₂ at doses of 0, 0.5, 1, or 2 mg/kg for 14 consecutive days. A second cohort (*n* = 36) was similarly allocated to six groups: control, Cd, 4-PBA, Cd + 4-PBA, CQ and Cd + CQ. All treatments were administered via daily intraperitoneal injection throughout the study. On day 14, blood and liver tissues were collected for analyses of liver function, hematological parameters, and histopathology. The expression of target factors was analyzed via qRT-PCR and Western blotting. Results indicate that Cd exposure causes liver injury and disordered hepatocyte morphology. ERS markers Grp78 and Caspase-12 and autophagy-related factors Beclin-1, Atg5, P62 and LC3 are upregulated at both mRNA and protein levels. The endoplasmic reticulum-phagy (ER-phagy) receptor FAM134B and the apoptosis effector cleaved Caspase3 are upregulated at the protein level. These findings indicate that Cd induces ERS, UPR activation, autophagy, reticulophagy, and apoptosis. The ERS inhibitor 4-PBA markedly attenuated Cd-induced hepatic injury. Compared with the Cd group, the Cd + 4-PBA group showed decreased serum ALT and AST levels, as well as reduced RBC, WBC, MCH, and MCV counts. At the molecular level, mRNA and protein expression of ER stress markers Grp78, Caspase-12, PERK, eIF2α, ATF4, IRE1α, JNK, and ATF6 were all downregulated. Protein levels of the ER-phagy receptor FAM134B and the apoptosis effector cleaved Caspase-3 were also decreased. The autophagy inhibitor CQ aggravated such injury. Compared with the Cd group, the Cd + CQ group showed increased serum ALT and AST levels, as well as elevated RBC, MCH, and MCV counts. At the protein level, expression of the autophagy-related factors P62 and LC3, and the apoptosis effector cleaved Caspase-3 was significantly increased. These results show that Cd-induced ERS activates autophagy and reticulophagy mainly via the PERK, IRE1α, and ATF6 pathways, and this adaptive response clears autophagic substrates to alleviate hepatocyte damage.

## 1 Introduction

Cd is a common toxic heavy metal (1). With the acceleration of industrialization, anthropogenic activities have become the primary source of environmental pollution. As a non-essential heavy metal with high cytotoxicity, Cd enters organisms via multiple routes—including ingestion, inhalation, and dermal absorption—where it readily bioaccumulates and undergoes trophic transfer, thereby posing significant threats to ecosystem integrity and human health.

The endoplasmic reticulum (ER) serves as a pivotal cellular hub that orchestrates protein synthesis, folding, post-translational modifications, and subsequent intracellular trafficking (2), consequently, maintaining ER homeostasis is paramount for preserving normal cellular physiological functions (3). Environmental toxins, redox imbalance, or impaired protein degradation can compromise ER function, resulting in the luminal accumulation of misfolded proteins and the subsequent onset of ERS. To cope with this stress, cells initiate a highly conserved adaptive response called the UPR (4), which was primarily mediated through three transmembrane signaling pathways: IRE1α, PERK, and ATF6 (5). These pathways sense ER stress and transmit signals to reduce global protein load (6), upregulate molecular chaperones, promote degradation of abnormal proteins, and induce autophagy, collectively aiming to restore ER homeostasis. However, when ERS persists beyond the capacity of adaptive UPR signaling, unresolved proteotoxicity disrupts intracellular homeostasis, triggering caspase-dependent apoptosis and contributing to hepatocyte loss.

Autophagy is a lysosome-dependent intracellular degradation process that maintains cellular homeostasis by selectively removing damaged organelles, misfolded proteins (7), and surplus or dysfunctional cytoplasmic constituents—and simultaneously provides recycled amino acids, lipids, and energy to sustain cellular metabolism (8). Autophagy is especially critical under stress conditions such as nutrient deprivation, oxidative stress, pathogen infection, and exposure to environmental toxicants. It functions as a key pro-survival mechanism that promotes cellular adaptation and sustains cell viability. Autophagy begins with the formation of a double-membrane structure called a phagophore (9). Autophagosomes gradually extend, engulfing target materials such as misfolded protein aggregates, aged mitochondria and other damaged organelles; they then fuse to form double-membrane vesicles known as autophagosomes (10). Following the fusion of the autophagosome with the lysosome, an autolysosome is formed. During this process, acidic hydrolases, including cathepsins, act on the captured material, breaking it down into simple molecules such as amino acids and fatty acids (11). These degradation products are subsequently transported back into the cytoplasm via transporters on the lysosomal membrane, where they are reused in biosynthetic pathways and energy metabolism. To monitor these dynamic processes, LC3, Beclin-1 and P62 are recognized as key marker proteins involved in the autophagy process (12, 13). Specifically, following phosphorylation, LC3 is recruited to the autophagosome membrane; levels of its phosphorylated form, LC3-II, are widely used as a reliable indicator of autophagic activity. Concurrently, Beclin-1 plays a central role in the nucleation stage of autophagosome formation and is a key regulator of autophagy initiation. Furthermore, P62, as a selective autophagy adaptor protein, mediates the targeted degradation of specific cargo by binding to ubiquitinated substrates via its ubiquitin-interacting domain, while simultaneously interacting with LC3 on the autophagosome membrane via its LC3-interacting region (LIR) (13–15).

Autophagy thus exhibits a dichotomous function, serving not only in cellular renewal and quality control under physiological states, but also modulating outcomes in pathological contexts: moderate activation facilitates cellular adaptation and survival, whereas dysregulated autophagy—whether excessive or insufficient—may precipitate cellular dysfunction or even cell death (16). Selective autophagy is orchestrated by specific autophagy receptor proteins, which identify and target specific substrates for degradation, thereby sustaining cellular quality control and functional homeostasis (17). These specialized proteins, termed selective autophagy receptors, facilitate substrate identification through a sophisticated network of molecular interactions that bridge ubiquitin-mediated labeling with the autophagic machinery. Specifically, substrate recognition in selective autophagy is predominantly governed by a complex interplay that bridges ubiquitin-mediated labeling with specific autophagy receptors. More precisely, autophagy receptors such as P62 engage ubiquitinated substrates through their ubiquitin-binding domains (UBDs) (18), which specifically recognize polyubiquitin chains (e.g., K48- or K63-linked) exposed on the cargo surface. Simultaneously, these receptors feature an LC3-interacting region (LIR)—characterized by the core consensus sequence W/YXXL/V (19), that mediates direct interaction with phosphatidylethanolamine-conjugated LC3 (LC3-II) anchored to the autophagosomal membrane (20). This bivalent binding mechanism tethers ubiquitinated cargo to the expanding autophagosome, thereby ensuring its selective sequestration and subsequent lysosomal degradation. This selective recognition mechanism extends to endoplasmic reticulum phagocytosis (ER-phagy), where FAM134B (also known as JK-1 or RETREG1)—a member of the Sequence Similarity 134 family—was identified as the pioneering receptor specifically mediating the selective degradation of ER fragments (21). FAM134B resides on the endoplasmic reticulum membrane and engages LC3 via its LIR domain, thereby driving the selective encapsulation and degradation of damaged or surplus ER fragments by autophagosomes (22, 23). Conversely, loss of FAM134B function compromises ER-phagy, resulting in the accumulation of aberrant ER components; this perturbation subsequently amplifies endoplasmic reticulum stress, ultimately precipitating hepatocellular injury (24). ERS and autophagy engage in a dynamic, bidirectional crosstalk. Specifically, ERS triggers autophagic initiation by activating distinct branches of the UPR, notably thePERK-eIF2α-ATF4 and IRE1-JNK signaling pathways (25). This mechanism facilitates the clearance of misfolded proteins and damaged organelles, thereby alleviating proteotoxic stress on the endoplasmic reticulum and restoring cellular homeostasis. However, when stress persists and exceeds the cell’s compensatory capacity, autophagy may switch from a protective mechanism to a pro-death signal, ultimately promoting apoptosis (26).

As a typical environmental toxicant, Cd has been shown to simultaneously activate both autophagy and apoptotic pathways. Therefore, in this study, a Cd-exposed rat model was established via intraperitoneal injection of CdCl₂, aiming to investigate the biological role and regulatory mechanisms of autophagy in Cd-induced hepatocyte injury. Furthermore, this study seeks to clarify the interplay among ERS, autophagy, and apoptosis, providing new theoretical insights and potential therapeutic targets for the prevention and intervention of Cd-induced hepatotoxicity (27).

## 2 Materials and methods

### 2.1 Reagents and materials

Cadmium chloride (≥99.99%, no. C116344) and 4-PBA (≥99%, no. P132032) were purchased from Aladdin Industrial Corporation (Shanghai, China). CQ (≥97%, no. C843545) was obtained from Macklin Biochemical Technology Co., Ltd. (Shanghai, China). All chemicals were stored at room temperature in a dry and light-protected environment. Stock solutions were freshly prepared in sterile saline or appropriate solvents before to use.

### 2.2 Animal experiments

The Henan Experimental Animal Center (Zhengzhou, China) supplied the SPF-grade male Sprague–Dawley rats used in this study (4 weeks old; 140–150 g body weight; License No. SCXK (Yu) 2020–0008). The experiment is divided into two parts. The detailed grouping of experimental animals is presented in Table 1. Doses were validated per Zhou et al. (28) and Sun et al. (29). The experiment is conducted in two sequential phases. The first phase is carried out initially, and after completion of this phase, including the monitoring and assessment of relevant endpoints, the second phase is initiated. In each phase, rats underwent a 7-day acclimatization period under standard laboratory conditions, followed by intraperitoneal injection of the drug. Each experimental phase lasted 21 days: the first phase was the Cd exposure phase, and the second phase was the pharmacological intervention phase. This standardized duration ensures uniform treatment cycles across groups and allows direct comparability of physiological outcomes. Rats were acclimated for one week under standard laboratory conditions with a 12-h light–dark cycle and maintained at an ambient temperature of 24 °C ± 2 °C. During the experiment, all animals were fed standard rodent chow and had free access to water. Rats were individually housed in polycarbonate cages, and body weight was recorded weekly. The experimental protocol was approved by the Animal Ethics Committee of Henan University of Science and Technology (approval no.: 2025022).

**Table 1:** Animal grouping and treatment regimen (*n* = 6).

	Group	Treatment regimen
Part 1	Control group	Equal volume of physiological saline (i.p.)
	0.5 mg/kg Cd group	0.5 mg/kg b.w. of CdCl₂ (i.p.)
	1 mg/kg group	1 mg/kg b.w. of CdCl₂ (i.p.)
	2 mg/kg group	2 mg/kg b.w. of CdCl₂ (i.p.)
Part 2	Control group	Equal volume of physiological saline (i.p.)
	Cd group	1 mg/kg b.w. of CdCl₂ (i.p.)
	4-PBA group	50 mg/kg b.w. of 4-PBA (i.p.)
	Cd + 4-PBA group	1 mg/kg b.w. of CdCl₂ (i.p.) and 50 mg/kg b.w. of 4-PBA (i.p.)
	CQ group	60 mg/kg b.w. of CQ (i.p.)
	Cd + CQ group	1 mg/kg b.w. of CdCl₂ (i.p.) and 60 mg/kg b.w. of CQ (i.p.)

### 2.3 Hepatotoxicity biochemical analysis

Specific commercial kits obtained from the Nanjing Jiancheng Bioengineering Institute (Nanjing, China) were utilized to assess serum alanine aminotransferase (ALT, no. C009-2-1) and aspartate aminotransferase (AST, no. C010-2-1) activities (30, 31).

### 2.4 Hematological parameters

Blood samples were collected from the femoral artery of rats using an anticoagulant tube and a blood collection needle. An automated hematology analyzer was subsequently employed to determine whole blood counts of white blood cells (WBC), red blood cells (RBC), hemoglobin (HGB), mean corpuscular hemoglobin (MCH), and mean corpuscular volume (MCV) across all experimental groups.

### 2.5 Histopathologic al analysis

Following fixation in 4% paraformaldehyde, liver tissues underwent dehydration, paraffin infiltration, and slicing. The resulting sections were stained with H&E to evaluate histopathological alterations via light microscopy (40×). For each kidney section, histopathological lesions are semi-quantitatively scored in at least 10 randomly selected fields per section at 200 × magnification. Evaluated features included: (i) cytoplasmic vacuolization and degeneration of tubular epithelial cells; (ii) flattening of tubular epithelium; (iii) hyaline cast formation; (iv) tubular dilatation; and (v) presence of cellular debris or proteinaceous material in the tubular lumen. Each feature was assigned a score of 0 (absent) or 1 (present). The total histopathology score ranged from 0 (no abnormalities) to 5 (maximal severity), with higher scores indicating more severe renal tubular injury (32).

### 2.6 Total RNA isolation and qRT-PCR

To obtain cDNA, total RNA was first extracted from rat livers using TRIzol, with purity and concentration confirmed by A260/A280 ratios and 1% agarose gel electrophoresis. Primers targeting specific genes were retrieved from NCBI, designed with Oligo 6.0 and Primer Premier 5.0. Details regarding the specific primer sequences utilized for real-time PCR can be found in Table 2 (33). We verified the specificity of all primer sequences and calculated relative mRNA levels using the 2^-ΔΔCT^ formula. Based on its uniform expression observed in initial trials and its proven track record in hepatic injury studies, *β -actin* was designated as the reference gene (34). During assay optimization, primers were rigorously assessed for both specificity and amplification efficiency. Negligible fluctuations in CT values were detected across all samples, confirming stable expression under the experimental conditions.

**Table 2:** RT-qPCR primers for target genes.

	Gene	Target gene sequences (5′ → 3′)
ERS-Related Genes	Grp78	F: ATGGTGTGGGAGATCCTGTTTTC R: ATGGTGTGGGAGATCCTGTTTTC
	*Caspase-12*	F: TCGGAGAAGGAGCGAGCTTA R: AGCTGTTTGTCGGAATTGGC
	*PERK*	F: ACAAGGCTGTCACTCAGGTG R: GCTAGGAGCCTTGGAGCAC
	*eIF2α*	F: TTTCCGGGACAAGATGGCG R: AAGTGTGGGGGTCCATTCAC
	*ATF4*	F: TGTTGGCGGGGGACTTAATG R: AAAGGCATCCTCCTTGCCG
	*IRE1α*	F: GCGCAGGTGCAATGACATAC R: CATGCAAACTTCCGTCCAGG
	*JNK*	F: TTGATTTTGGACTGGCGAGGA R: TTGTGTGCTCCCTCTCATCTAA
	*ATF6*	F: TCATGGACCAGGTGAAGACT R: GGGGCTCCATATGTCTGACTC
Autophagy-related genes	*Beclin-1*	F: GGAGATGTTGGAGCAAATGGTT R: GTCATGGACCAGGTGAAGACT
	*Atg5*	F: ATGCAGTTGAGGCTCACTTTATGTC R: TGGAGGGTATTCCATGAGTTTCC
	*P62*	F: TATTACAGCCAGAGTCAAGG R: CATCATACAGAAGCCAGAATG
	*LC3*	F: ACGGCTTCCTGTACATGGTC R: GTGGGTGCCTACGTTCTGAT
	*IRE1α*	F: GCGCAGGTGCAATGACATAC R: CATGCAAACTTCCGTCCAGG
	*JNK*	F: TTGATTTTGGACTGGCGAGGA R: TTGTGTGCTCCCTCTCATCTAA
	*β-Actin*	F: CACCCGCGAGTACAACCTT R: TCATCCATGGCGAACTGGTG

### 2.7 Western blot

Liver tissues were collected and homogenized on ice using RIPA lysis buffer (Beyotime, P0013B, Shanghai, China), which was freshly supplemented with a protease inhibitor cocktail to prevent protein degradation. Homogenization were performed using a handheld electric homogenizer until complete tissue disruption was achieved. To ensure complete solubilization, lysates were vortexed vigorously at 4 °C for 30 min and then centrifugation was performed at 12000 r for 10 min (4 °C). Total protein was collected from the supernatant, and its concentration was measured via a BCA Protein Assay Kit (Beyotime, P0013) following the supplier’s guidelines. Protein samples were mixed with 5 × reduced loading buffer at a 4:1 ratio and thermally denatured in a boiling water bath for 15 min. The treated aliquots were then stored at −20 °C until further use. Subsequently, protein lysates were separated via 12% SDS-PAGE, transferred onto PVDF membranes, and blocked with 5% skim milk in TBST for 2 h.

Upon completion of blocking, an overnight incubation at 4 °C was performed using primary antibodies directed against critical regulators of ERS, autophagy, and apoptosis, which included: Grp78, Caspase-12, Beclin-1, Atg5, P62, LC3, PERK, eIF2α, ATF4, IRE1α, JNK, ATF6, cleaved Caspase-3, and *β*-actin. Following three washes with TBST, membranes were incubated for 2 h at room temperature with HRP-conjugated goat anti-rabbit or anti-mouse IgG secondary antibodies. Membranes were washed three times with TBST (10 min each), immunoreactive bands were visualized using ECL reagent (Beyotime, P0018FS). Images were captured in a darkroom using a chemiluminescent imaging system. Each sample was run in triplicate to ensure reproducibility. ImageJ software was used to quantify band intensities, and target protein levels were normalized against *β*-actin. Primary antibodies were diluted in antibody dilution buffer according to the manufacturer’s instructions (35). The working solutions of secondary antibodies were generated by diluting them in TBST with 5% non-fat milk at room temperature just before the incubation step. Details of the required antibodies and corresponding dilution ratios are provided in Table 3.

**Table 3:** Summary of primary and secondary antibodies utilized in western blot analysis.

Antibody name	Dilution ratio	Catalog number	Manufacturer
Grp78	1:5000	PA1815	Boster
Caspase-12	1:12000	5528-1-AP	ABclonal
PERK	1:2000	ab229912	Proteintech
eIF2α	1:5000	ab169528	Abcom
ATF4	1:3000	A18687	Proteintech
IRE1α	1:3000	27528-1-AP	Proteintech
JNK	1:2000	WL01295	Wanleibio
ATF6	1:3000	24169-1-AP	Proteintech
Beclin-1	1:5000	11306-1-AP	Proteintech
Atg5	1:1000	10181-2-AP	Proteintech
P62	1:3000	84826-1-RR	Proteintech
LC3	1:2000	14600-1-AP	Proteintech
Cleaved caspase-3	1:1000	WL01992	Wanleibio
β-Actin	1:10000	AC004	Proteintech
Horseradish peroxidase-conjugated anti—mouse antibody	1:12000	HA1006	HUABIO
Horseradish peroxidase-conjugated anti—rabbit antibody	1:12000	HA1001	HUABIO

### 2.8 Statistical analyses

Data were analyzed using SPSS 22.0 software. Group comparisons were performed using one-way analysis of variance (ANOVA) followed by the LSD post-hoc test. Graphs were generated with GraphPad Prism 7. All results are expressed as mean ± standard error of the mean (SEM). Statistical significance was defined as *p* < *0.05*, and *p* < *0.01* indicated highly significant differences (36).

## 3 Results

### 3.1 Effects of Cd exposure on body weight and liver function in rats

**Figure 1:**
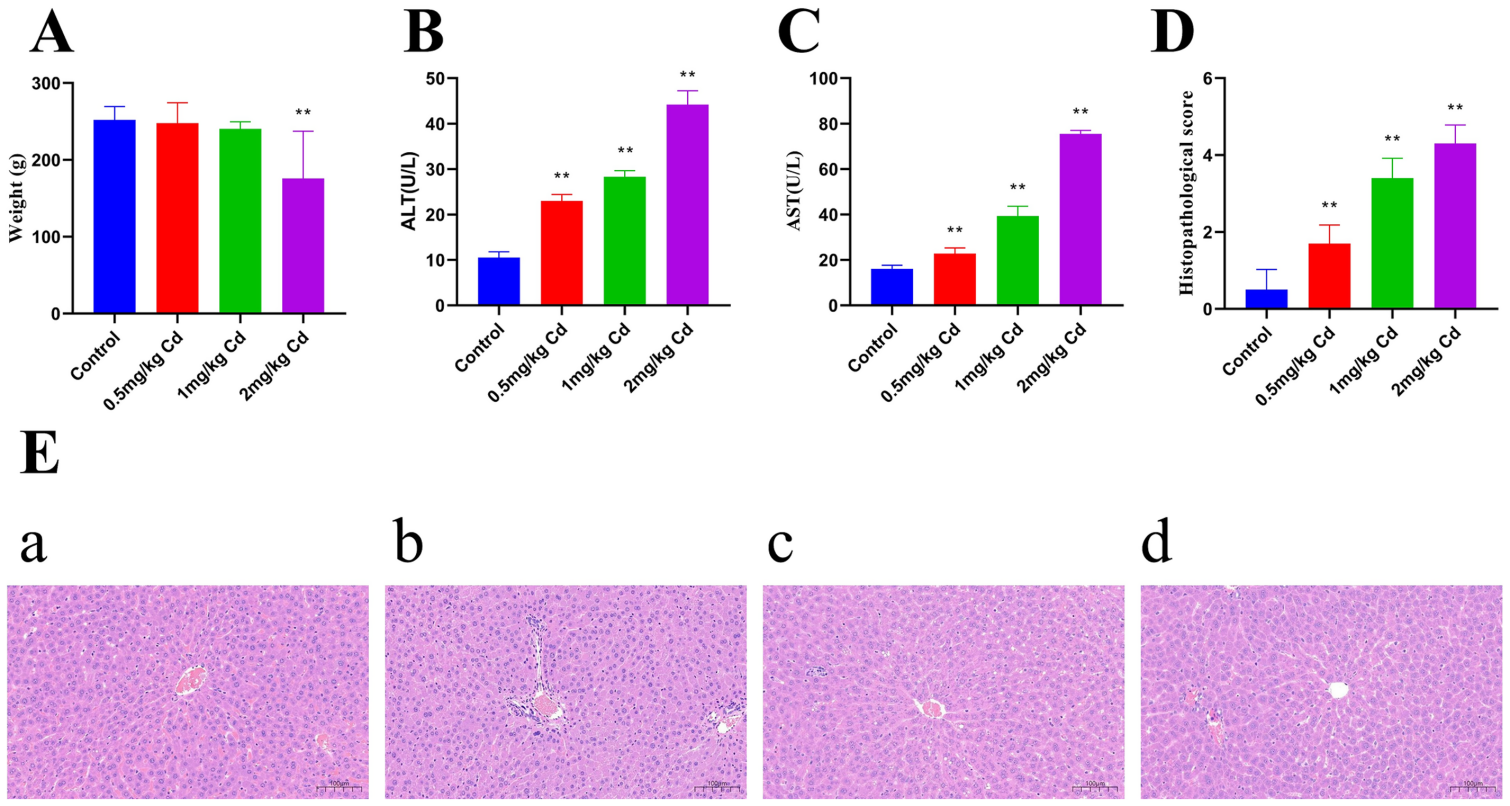
Effects of Cd exposure on body weight, liver function, and hepatic histopathology in rats. **(A)** body weight; **(B)** serum ALT activity; **(C)** serum AST activity; **(D)** histopathological score; **(E) (a–d)** histopathological sections (40×). **(a)** Control group; **(b)** 0.5 mg/kg Cd group; **(c)** 1 mg/kg group; **(d)** 2 mg/kg group. *p < *0.05* indicates a significant difference; ^**^p < 0.01 indicates a highly significant difference.

As shown in Figures 1A–D, compared with the control group, body weight was extremely significantly decreased in the 2 mg/kg Cd group (*p* < *0.01*). ALT and AST levels were extremely significantly increased (*p* < *0.01*) in different Cd concentration groups, showing a dose-dependent effect. As shown in Figure 1E, the central vein in the control group exhibited regular morphology, and hepatocytes were arranged orderly without vacuolation or necrosis [Figure 1E(a)]. In the 0.5 mg/kg Cd group, a few small vacuolated areas appeared around the central vein, and mild sinusoidal dilation was observed [Figure 1E(b)]. In the 1 mg/kg Cd group, the vacuolated areas expanded, hepatocyte arrangement became slightly disordered, and sinusoidal dilation was more pronounced [Figure 1E(c)]. In the 2 mg/kg Cd group, extensive vacuolation was observed throughout the tissue, large vacuoles appeared adjacent to the central vein, and overall hepatic architecture was severely disrupted [Figure 1E(d)].

### 3.2 Cd induces ERS and activates the UPR pathways PERK, IRE1α, and ATF6

**Figure 2:**
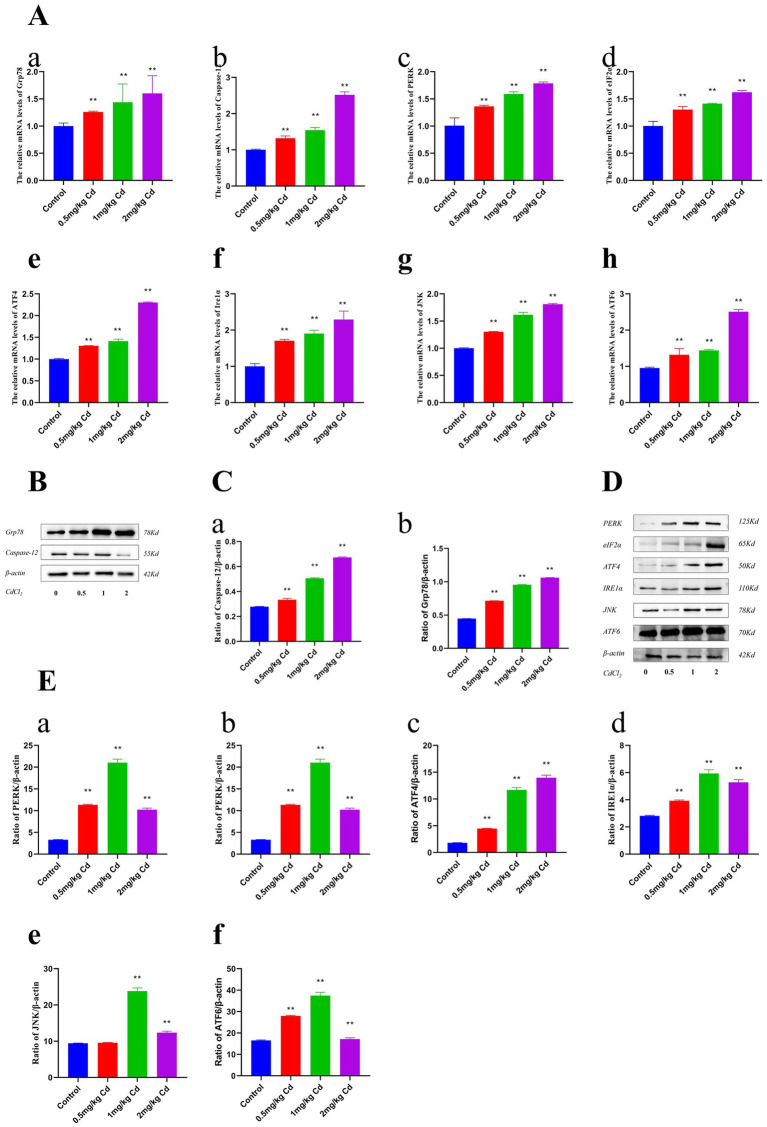
Cd induces ERS and activates the PERK, IRE1α, and ATF6 signaling pathways in rats. **(A)** mRNA expression levels of related factors. **(a)** Grp78; **(b)** Capase12; **(c)** PERK; **(d)** eIF2α; **(e)** ATF4; **(f)** IRE1α; **(g)** JNK; **(h)** ATF6. **(B)** Blots for Grp78 and Capase12. **(C)** Protein expression levels of related factors. **(a)** Grp78; **(b)** Capase12. **(D)** Blots for PERK, eIF2α, ATF4, IRE1α, JNK and ATF6. **(E)** Protein expression levels of related factors. **(a)** PERK; **(b)** eIF2α; **(c)** ATF4; **(d)** IRE1α; **(e)** JNK; **(f)** ATF6. *β*-actin served as an internal reference and quantitative analysis of band intensities was performed using ImageJ software. Compared with the control group *p < 0.05 indicates a significant difference; **p < 0.01 indicates a highly significant difference.

As shown in Figure 2, compared with the control group, mRNA and protein expression levels of ERS markers, including GRP78, Caspase-12, PERK, eIF2α, ATF4, IRE1α, JNK, and ATF6 were highly significantly increased in the livers of cadmium-treated rats (*p* < *0.01*).

### 3.3 Cd induces autophagy, reticulophagy, and apoptosis in rat hepatocytes

**Figure 3:**
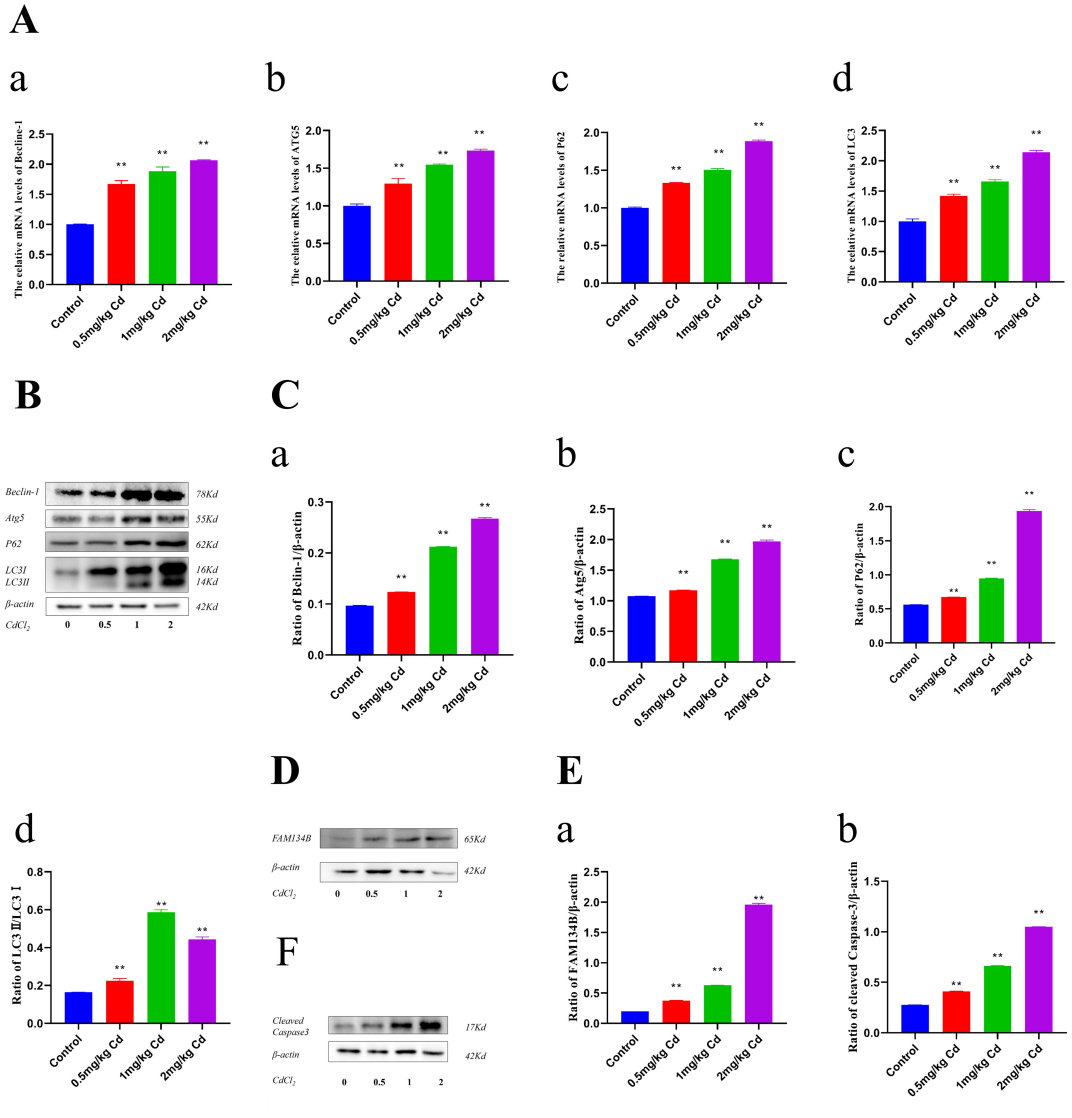
Cd exposure induces autophagy, reticulophagy, and apoptosis in rat hepatocytes. **(A)** mRNA expression levels of related factors. **(a)** Beclin-1; **(b)** Atg5; **(c)** P62; **(d)** LC3. **(B)** Western blot images for Beclin-1, Atg5, p62, and LC3. **(C)** Protein expression levels of related factors. **(a)** Beclin-1; **(b)** Atg5; **(c)** P62; **(d)** LC3; **(D)** Blots for FAM134B. **(E)** cleaved Caspase-3. **(F)** Protein expression levels of related factors. **(a)** FAM134B; **(b)** cleaved Caspase-3. *β*-actin served as an internal reference and quantitative analysis of band intensities was performed using ImageJ software. Compared with the control group *p < 0.05 indicates a significant difference; **p < 0.01 indicates a highly significant difference.

As shown in Figure 3, the mRNA and protein expression of the autophagy regulator Beclin-1, Atg5, P62, and LC3 were significantly increased in rat livers after Cd treatment (*p* < *0.01*). Protein expression levels of the ER-phagy receptor FAM134B and the apoptosis effector cleaved Caspase-3 were highly significantly increased (*p* < *0.01*).

### 3.4 Effects of 4-PBA and CQ intervention on body weight, liver function, and hepatic histopathology in Cd-exposed rats

**Figure 4:**
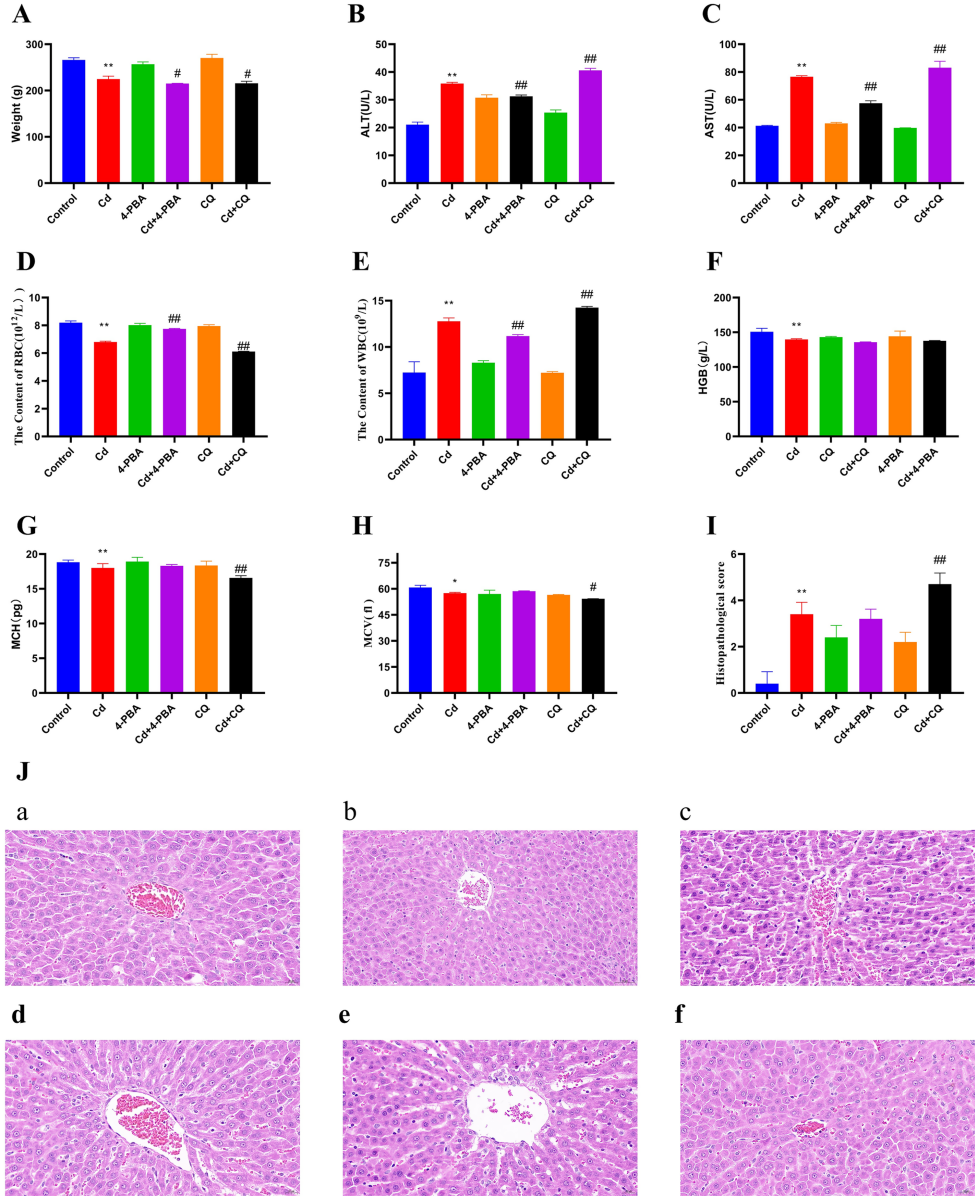
Effects of CQ and 4-PBA on body weight, liver function, hematological parameters, and hepatic histopathology in Cd-exposed rats. **(A)** Weight; **(B,C)** Liver function; **(B)** ALT; **(C)** AST; **(D–H)** Hematological parameters; **(D)** RBC; **(E)** WBC; **(F)** HGB; **(G)** MCH; **(H)** MCV; **(I)** Histopathological score; **(J) (a–f)** Histopathological sections (40×). **(a)** Control group; **(b)** Cd group; **(c)** 4-PBA group; **(d)** Cd + 4-PBA group. **(e)** CQ group; **(f)** Cd + CQ group. Compared with the control group *p < 0.05 indicates a significant difference; **p < 0.01 indicates a highly significant difference. Compared with the Cd group, #*p* < 0.05 indicates a significant difference; ##*p* < 0.01 indicates a highly significant difference.

As shown in Figure 4, compared with the control group, the Cd group exhibited a highly significant (*p* < *0.01*) decrease in body weight and highly significant (*p* < *0.01*) increases in serum ALT and AST levels; blood parameters showed significant or highly significant (*p* < *0.01* or *p* < *0.05*) decreases in RBC, HGB, MCH, and MCV, and a highly significant increase (*p* < *0.01*) in WBC. Compared with the Cd group, the Cd + 4-PBA group showed a significant (*p* < *0.01*) decrease in body weight, highly significant (*p* < *0.01*) decreases in ALT and AST, a highly significant (*p* < *0.01*) increase in RBC, and a highly significant (*p* < *0.01*) decrease in WBC. In the Cd + CQ group, body weight decreased significantly; ALT and AST levels increased highly significantly (*p* < *0.01*); and blood parameters showed a significant (*p* < *0.01*) decrease in RBC, and highly significant increases in WBC, MCH, and MCV.

As shown in Figure 4J, histopathological examination revealed that control group hepatocytes were neatly arranged in radial cords around central veins, with normal morphology, clear nuclei, uniform cytoplasmic staining, and intact hepatic lobules — indicating normal liver physiology. The Cd group showed moderate changes, including hepatocyte swelling and cytoplasmic vacuolation, with partial structural disarray. The Cd + 4-PBA group showed reduced disorganization compared with the Cd group, but hepatocyte architecture and lobular structure remained incompletely restored. The Cd + CQ group exhibited aggravated disruption, with more extensive swelling, degeneration, and focal necrosis.

### 3.5 Cd-induced ERS mediates autophagy activation in rat hepatocytes

**Figure 5:**
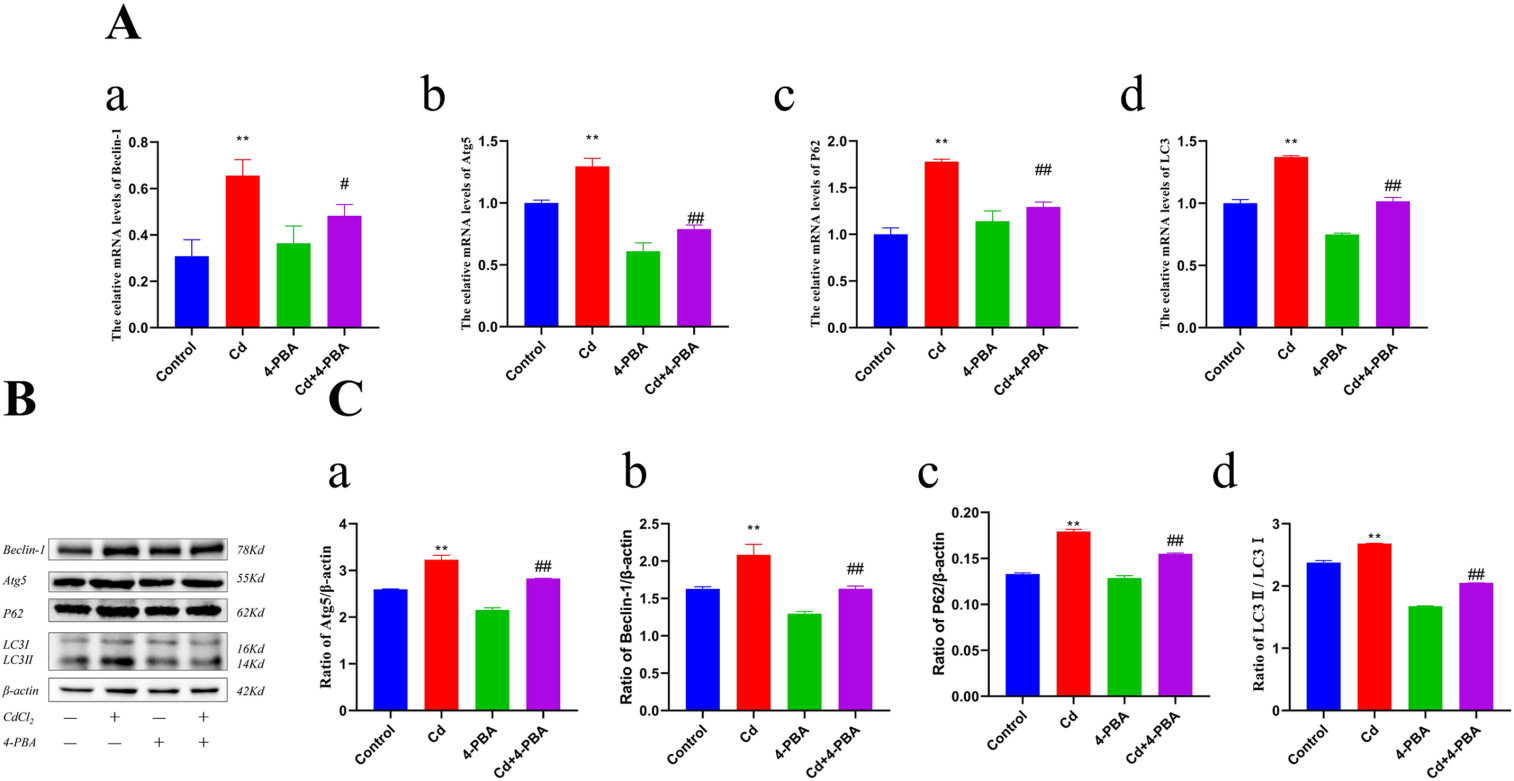
Effect of 4-PBA mediated ERS inhibition on autophagy. **(A)** mRNA expression levels of autophagy-related factors. **(a)** Beclin-1; **(b)** P62; **(c)** Atg5; **(d)** LC3. **(B)** Blots for Beclin-1, P62, Atg5, and LC3. **(C)** Protein expression levels of autophagy-related factors. **(a)** Beclin-1; **(b)** P62; **(c)** Atg5; **(d)** LC3. *β*-actin served as an internal reference and quantitative analysis of band intensities was performed using ImageJ software. Compared with the control group *p < 0.05 indicates a significant difference; **p < 0.01 indicates a highly significant difference. Compared with the Cd group, #*p* < 0.05 indicates a significant difference; ##*p* < 0.01 indicates a highly significant difference.

As shown in Figure 5, compared with the control group, Cd exposure significantly upregulated mRNA and protein expression of the autophagy-related factors Beclin-1, Atg5, p62, and LC3 (*p* < *0.01*). In contrast, Cd + 4-PBA group markedly downregulated the expression of these factors at both mRNA and protein levels (*p* < *0.01*).

### 3.6 Cd-induced ERS in rat hepatocytes mediates autophagy activation via the PERK, IRE1α, and ATF6 pathways

**Figure 6:**
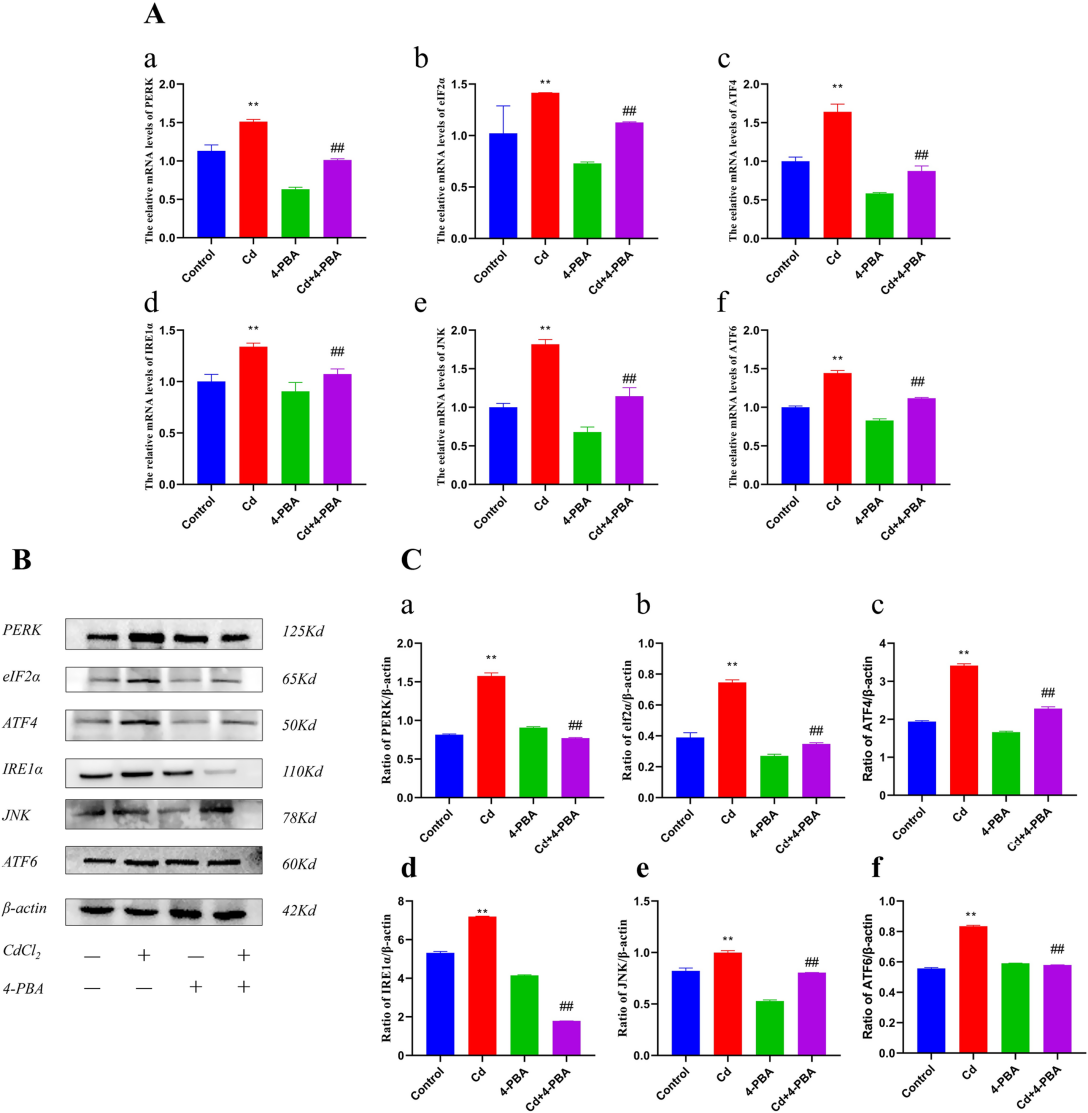
4-PBA treatment modulates Cd-induced protein expression of the ERS-related PERK, IRE1α, and ATF6 pathways in rat hepatocytes. **(A)** mRNA expression levels of related factors **(a)** PERK; **(b)** eIF2α; **(c)** ATF4; **(d)** IRE1α; **(e)** JNK; **(f)** ATF6. **(B)** Blots for PERK, eIF2α, ATF4, IRE1α, JNK, and ATF6. **(C)** Protein expression levels of the **(a)** PERK; **(b)** eIF2α; **(c)** ATF4; **(d)** IRE1α; **(e)** JNK; **(f)** ATF6. *β*-actin served as an internal reference and quantitative analysis of band intensities was performed using ImageJ software. Compared with the control group *p < 0.05 indicates a significant difference; **p < 0.01 indicates a highly significant difference. Compared with the Cd group, #*p* < 0.05 indicates a significant difference; ##*p* < 0.01 indicates a highly significant difference.

In the 4-PBA intervention experiment Figure 6, Cd-treated rats still exhibited marked elevation in the expression of these UPR-related molecules relative to controls (*p* < *0.01*). However, compared with the Cd group, the Cd + 4-PBA group showed a significant downregulation of PERK, eIF2α, ATF4, IRE1α, JNK, and ATF6 at both mRNA and protein levels (*p* < *0.01*), suggesting that 4-PBA effectively suppresses the activation of the PERK/eIF2α/ATF4, IRE1α/JNK, and ATF6 signaling pathways by alleviating ERS, thereby modulating aberrant autophagy induction.

### 3.7 Cd-induced ERS in rat hepatocytes triggers selective reticulophagy

**Figure 7:**
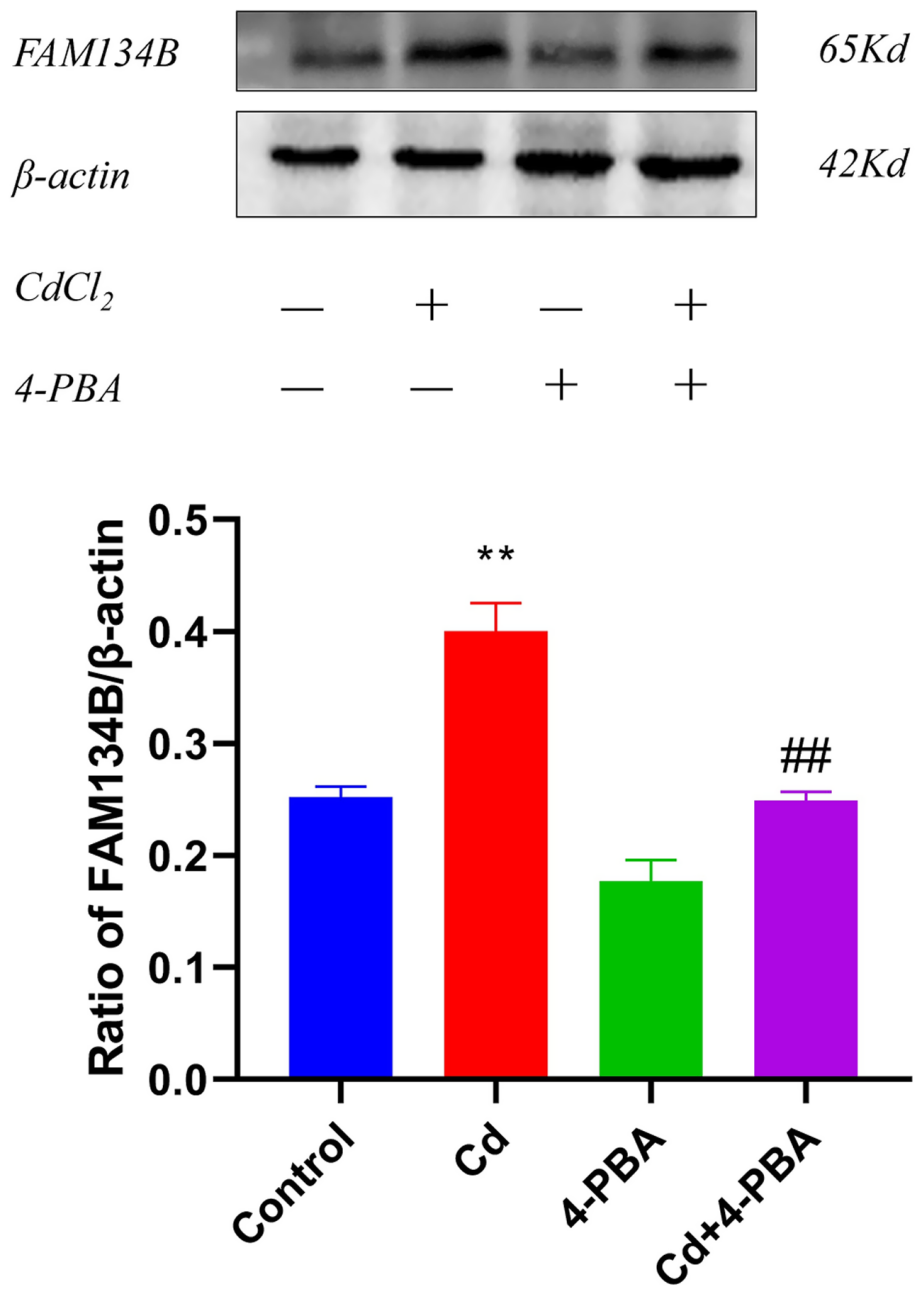
Effect of 4-PBA treatment on reticulophagy. *β*-actin served as an internal reference and quantitative analysis of band intensities was performed using ImageJ software. Compared with the control group *p < 0.05 indicates a significant difference; **p < 0.01 indicates a highly significant difference. Compared with the Cd group, #*p* < 0.05 indicates a significant difference; ##*p* < 0.01 indicates a highly significant difference.

As shown in Figure 7, FAM134B protein expression was highly significantly increased in the Cd group compared with the control group (*p* < *0.01*), and was highly significantly decreased in the Cd + 4-PBA group compared with the Cd group (*p* < *0.01*).

### 3.8 Effects of 4-PBA and CQ intervention on ERS, autophagy, and Cd-induced hepatocyte apoptosis in rats

**Figure 8:**
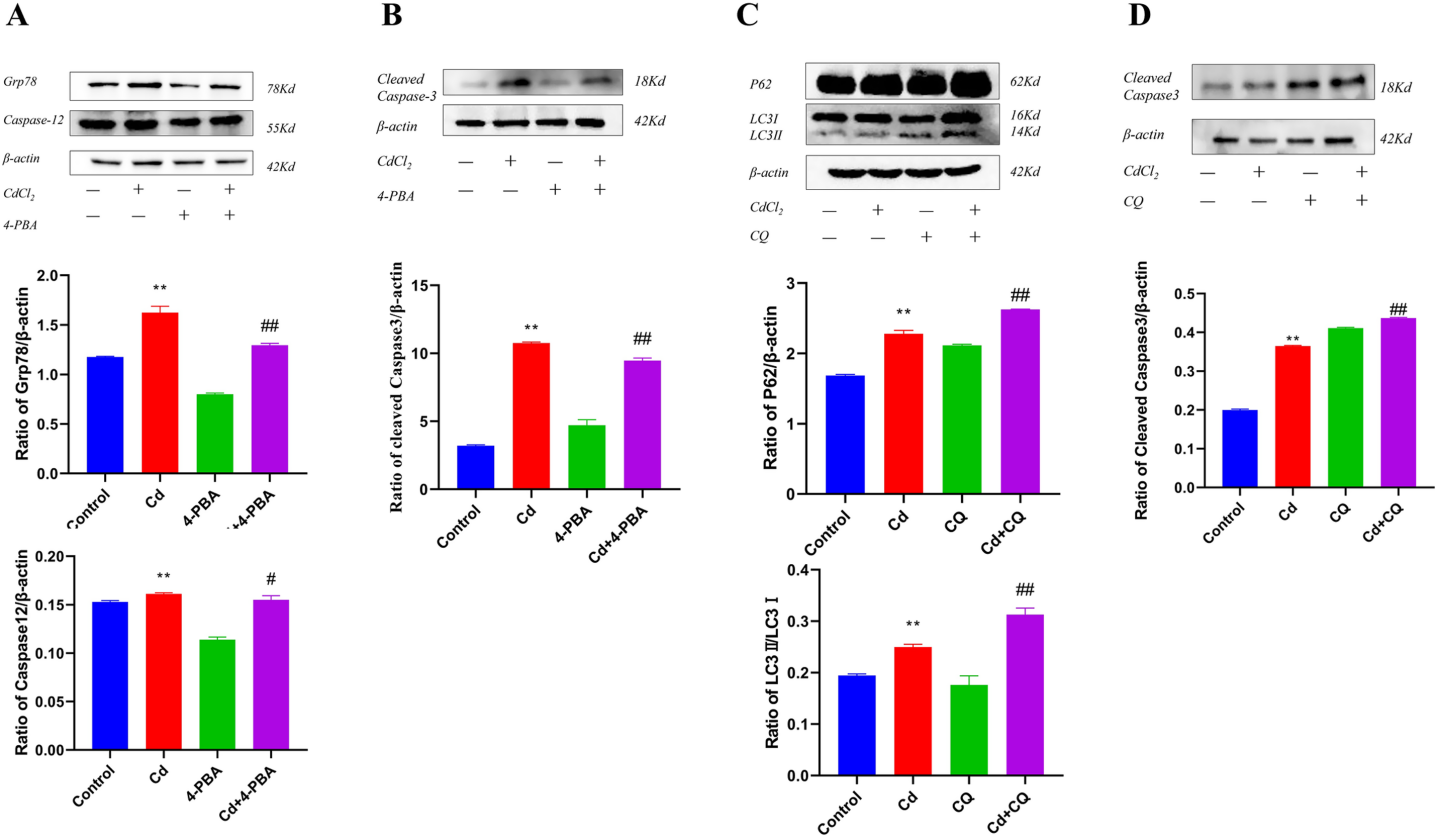
The impact of ERS and autophagy on apoptosis. **(A)** Western blot images and quantitative analysis of Grp78 and Caspase-12. **(B)** Western blot images and quantitative analysis of cleaved Caspase-3 after 4-PBA treatment. **(C)** Western blot images and quantitative analysis of P62 and LC3. **(D)** Western blot images and quantitative analysis of cleaved Caspase-3 after CQ treatment. *β*-actin served as an internal reference and quantitative analysis of band intensities was performed using ImageJ software. Compared with the control group *p < 0.05 indicates a significant difference; **p < 0.01 indicates a highly significant difference. Compared with the Cd group, #*p* < 0.05 indicates a significant difference; ##*p* < 0.01 indicates a highly significant difference.

**Figure 9:**
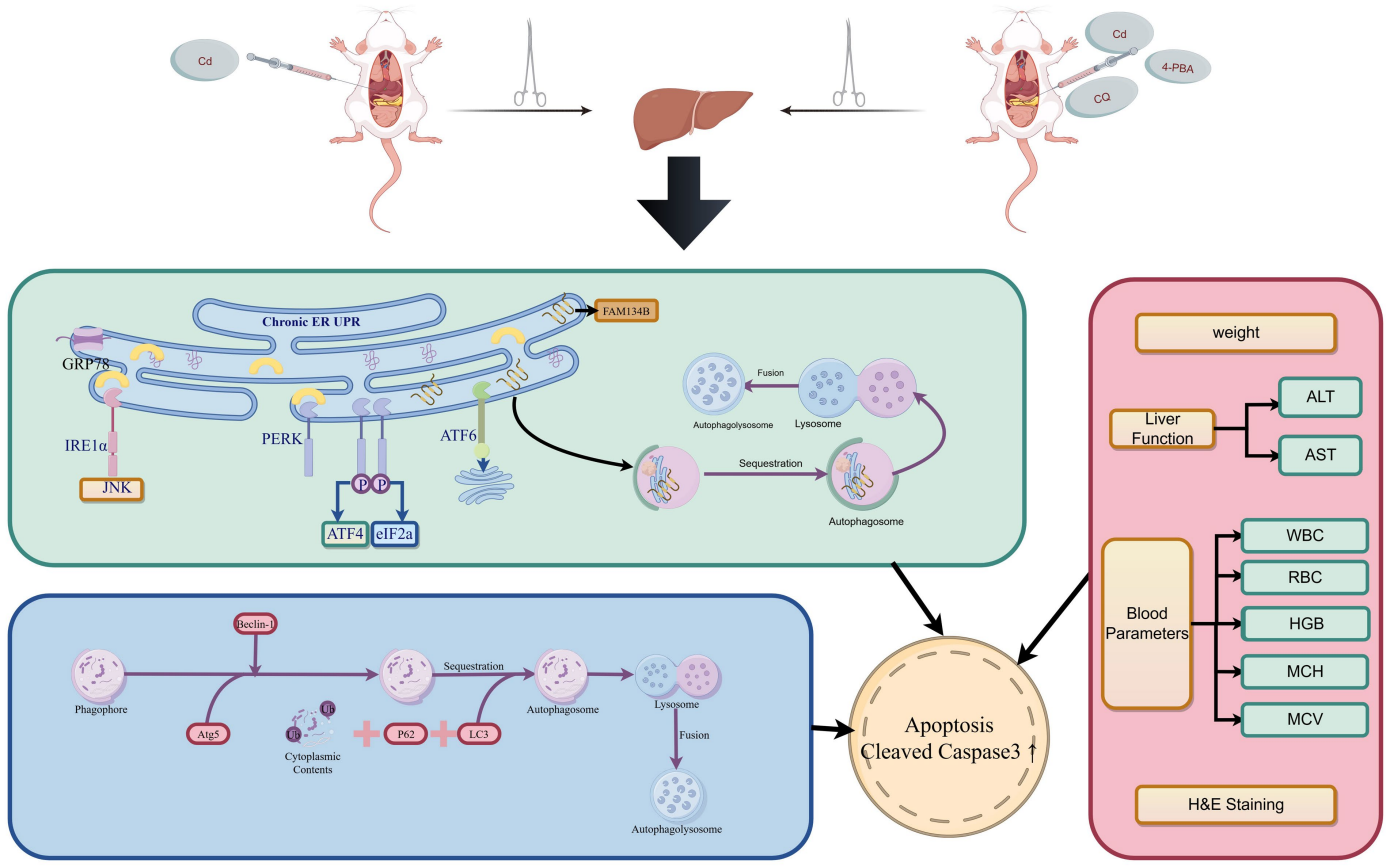
Mechanism of endoplasmic reticulum stress–mediated autophagy in Cd-induced liver injury in rats.

After 4-PBA intervention, as shown in Figures 8A,B, protein expression levels of Grp78, Caspase-12, and cleaved Caspase-3 in the Cd group were highly significantly increased compared with the control group (*p* < *0.01*). Compared with the Cd group, protein expression levels of Grp78, Caspase-12, and cleaved Caspase-3 in the Cd + 4-PBA group were highly significantly or significantly decreased (*p* < *0.01* or *p* < *0.05*). After CQ intervention, as shown in Figures 8C,D, protein expression levels of p62, LC3, and cleaved Caspase-3 in the Cd group were highly significantly increased compared with the control group (*p* < *0.01*). Compared with the Cd group, protein expression levels of p62, LC3, and cleaved Caspase-3 in the Cd + CQ group were highly significantly increased (*p* < *0.01*) (see Figure 9).

## 4 Discussion

Body weight in rats is one of the key indicators for assessing their overall health status and the effects of toxicant exposure. Weight gain in rats in the Cd-treated group was markedly retarded, exhibiting a dose-dependent decline, consistent with the findings of Chen et al. (37). The liver serves as the primary metabolic organ in animals, with ALT and AST constituting core serum markers of hepatic function. These enzymes reside within the cytoplasm and mitochondria of hepatocytes, and elevated serum levels serve as sensitive indicators of hepatocyte membrane damage. Alfwuaires et al. demonstrated that Cd exposure induces liver injury and functional impairment (38). The results of HE staining and liver function tests revealed that, compared with the control group, Cd treatment led to a significant increase in ALT and AST activity, with a progressive trend observed as the toxicant dose increased (39). Cd exposure triggers multi-layered stress responses in hepatocytes. This study demonstrates that Cd induces ERS, as evidenced by marked upregulation of ERS markers Grp78 and Caspase-12, and concurrent activation of all three canonical branches of the UPR: the PERK/eIF2α/ATF4 axis, the IRE1α/JNK axis, and the ATF6 pathway—indicating severe disruption of ER proteostasis. Accumulation of autophagy-related proteins Beclin-1, Atg5, and LC3-II, along with impaired P62 degradation (despite elevated total p62 protein levels—a hallmark of autophagic flux initiation rather than complete blockade), together with significant upregulation of the ER-phagy receptor FAM134B, indicate that Cd not only induces general autophagy but also specifically activates selective reticulophagy to eliminate damaged ER fragments. However, under sustained or excessive stress, the apoptosis effector cleaved Caspase-3 is robustly activated, suggesting that protective autophagy and reticulophagy ultimately fail to compensate, leading hepatocytes toward programmed cell death. Grp78, as the classic molecular chaperone of the ERS, dissociates from the UPR sensor upon accumulation of misfolded proteins and activates downstream signalling pathways (40). Its elevated expression reflects an imbalance in ER protein homeostasis (41). Caspase-12 is an apoptotic protein localized to the endoplasmic reticulum, specifically responding to ERS stimuli and participating in apoptosis initiation (42). Its elevated expression indicates that ERS has transitioned from an adaptive response to a pro-death signalling pathway. The upregulation of Beclin-1 and Atg5, key regulators of autophagy initiation and autophagosome formation, indicates that the autophagy process has been activated (43). The LC3-II/LC3-I ratio showed a marked increase, further confirming heightened autophagosome formation. However, p62 protein levels did not decrease but instead rose. This, combined with the accumulation of LC3-II, suggests that although autophagy was initiated, the autophagic flux may have been impeded. Consequently, late autophagic degradation function was impaired, preventing the effective clearance of abnormal proteins and damaged organelles. Research by Xueru Wang et al. indicates that molybdenum and/or Cd-induced autophagy may be associated with the cross-activation of Nrf2-mediated antioxidant defence responses and ERS in duck liver, with molybdenum and Cd synergistically exacerbating hepatic toxic injury (43). Collectively, these findings demonstrate that Cd exposure effectively induces ER stress in hepatocytes, thereby comprehensively activating all three UPR branches and coordinately triggering autophagy, selective reticulophagy, and Caspase-dependent apoptosis—processes that collectively drive the onset and progression of Cd-induced liver injury.

Cd exposure not only activates stress defence mechanisms such as the ERS and autophagy, but also triggers irreversible apoptotic programmes. ERS-mediated autophagy activation in Cd-induced hepatic injury: functional roles of the ERS–autophagy axis. In this study, the autophagy inhibitor CQ and the ERS inhibitor 4-PBA were used to systematically investigate the effects of targeted modulation of either ERS or autophagy on Cd-induced hepatotoxicity — specifically, whether such intervention exacerbates or alleviates liver injury. Compared with the control group, the Cd group exhibited a highly significant decrease in body weight, highly significant increases in serum ALT and AST levels, and significant or highly significant decreases in RBC, HGB, MCH, and MCV — indicating impaired erythropoiesis and microcytic/hypochromic red blood cell changes; WBC count was highly significantly elevated, suggesting systemic inflammation. Compared with the Cd group, the Cd + 4-PBA group showed a significant decrease in body weight— reflecting partial mitigation of growth inhibition; serum ALT and AST levels were highly significantly reduced, indicating marked attenuation of hepatocellular injury; RBC count was highly significantly increased, and WBC count was highly significantly decreased — consistent with improved erythropoietic function and suppressed inflammatory response. MCH and MCV remained unchanged or showed no statistically significant recovery relative to the Cd group (i.e., no normalization), implying that 4-PBA alleviated acute hepatotoxicity and inflammation but did not fully reverse Cd-induced erythrocyte morphological alterations. In contrast, the Cd + CQ group exhibited a significant decrease in body weight (worsening relative to Cd alone); highly significant further elevations in ALT and AST—confirming aggravated hepatocyte damage upon autophagy blockade; a significant decrease in RBC (exacerbating anemia); and highly significant increases in WBC, MCH, and MCV—suggesting intensified inflammatory activation, compensatory erythropoietic stress, and possible macrocytic shifts secondary to oxidative damage or impaired heme synthesis. Collectively, these results demonstrate that 4-PBA–mediated inhibition of ERS effectively ameliorates Cd-induced liver injury and systemic inflammation but has limited efficacy in restoring erythrocyte indices such as MCH and MCV, whereas chloroquine–mediated inhibition of autophagy exacerbates both hepatic dysfunction and hematological disturbances — highlighting the critical protective role of functional autophagy in mitigating Cd toxicity.

ERS arises from protein folding and metabolic imbalances triggered under conditions such as protein overload, oxidative stress, calcium imbalance, and nutritional deficiency (44). Autophagy constitutes a process within cells whereby proteins and organelles undergo extensive degradation dependent upon lysosomes (44). However, the relationship between the two remains unclear. Following 4-PBA treatment, which suppresses ERS, the expression of Beclin-1 and Atg5 was significantly reduced at both the mRNA and protein levels. The LC3-II to LC3-I ratio decreased, and p62 protein levels declined markedly (45). These changes reflect attenuated autophagosome formation and restored autophagic flux. The coordinated downregulation of Beclin-1, Atg5, LC3-II, and P62 indicates that autophagy activation is not an independent response but a downstream consequence of ERS. Thus, Cd-induced ERS acts as the primary trigger that drives autophagy through Beclin-1 and Atg5 dependent pathways, with LC3 processing and P62 degradation serving as functional readouts of this regulated process.

Cd induces ERS and concurrently activates the UPR signaling pathways; it also triggers autophagy. Specifically, Cd exposure enhances phosphorylation of PERK and its downstream effectors eIF2α and ATF4; increases phosphorylation of IRE1α; and promotes proteolytic cleavage of ATF6, leading to nuclear translocation of the active ATF6-N fragment (46). These changes confirm robust and coordinated activation of the PERK, IRE1α, and ATF6 pathways. Critically, this UPR activation is temporally and mechanistically coupled to autophagy induction. The PERK–eIF2α–ATF4 axis directly upregulates transcription of Atg5 and Beclin-1, thereby promoting autophagosome nucleation and formation (46). The IRE1α pathway supports phagophore expansion and membrane elongation by modulating expression of lipid synthesis and membrane remodeling genes. ATF6 activation not only induces ER chaperones such as Grp78 to alleviate proteotoxic load but also contributes to the transcriptional regulation of autophagy-related genes. Importantly, 4-PBA–mediated suppression of ERS markedly attenuates activation of all three UPR arms—and concomitantly reduces Beclin-1 expression and LC3-II accumulation. This parallel inhibition demonstrates that autophagy initiation in Cd-exposed hepatocytes is dependent on intact signaling through the PERK, IRE1α, and ATF6 pathways. FAM134B, the first identified ER-resident selective autophagy receptor (47), was significantly upregulated in Cd-exposed rat hepatocytes. Under Cd exposure, FAM134B protein expression was significantly upregulated — a change that closely paralleled the activation of ERSmarkers Grp78 and CHOP, as well as the phosphorylation/activation of PERK, IRE1α, and proteolytic cleavage of ATF6 (48). Upon inhibition of ERS by 4-PBA, FAM134B expression was subsequently downregulated. This suggests its activation depends on the ongoing presence of ERS. Mechanistically, upon ERS activation, the molecular chaperone Grp78 dissociates from FAM134B, releasing its conformation inhibition and exposing its LC3-interacting domain. This enables specific binding to LC3-II localised on autophagosome membranes. It forms the FAM134B LC3 protein complex (49). This complex mediates selective engulfment and targeted transport of damaged ER fragments. Consistent with this, Cd exposure increased both FAM134B levels and LC3-II accumulation, indicating active reticulophagy initiation (23). Together, these findings establish FAM134B as a key molecular link through which Cd-induced ERS directly drives selective ER turnover.

Meanwhile, impairment of autophagic degradation significantly affects cellular fate. 4-PBA intervention effectively suppressed ERS, as evidenced by markedly reduced protein expression of Grp78 and Caspase-12 in the Cd + 4-PBA group compared with the Cd group; concomitant attenuation of autophagy and decreased accumulation of autophagic substrates were associated with reduced cleaved Caspase-3 expression, indicating that alleviating substrate accumulation mitigates Cd-induced injury in rat hepatocytes. In contrast, CQ blocks autophagosome-lysosome fusion without affecting upstream signalling pathways (50). Results showed that chloroquine treatment alone or in combination with Cd led to significant accumulation of LC3-II and p62 proteins, most prominently in the Cd + CQ group — a hallmark of impaired autophagic flux. This confirms that Cd induces autophagy initiation; when the degradation pathway is blocked by chloroquine, unprocessed autophagic substrates accumulate persistently, further exacerbating cellular burden and accompanied by further upregulation of ER stress markers including Grp78 and Caspase-12. This establishes a vicious cycle: ERS mediates autophagy initiation, but failure to complete autophagic degradation leads to toxic substrate accumulation, which in turn intensifies ERS. It should be acknowledged that the intraperitoneal injection of CdCl₂ employed in this study does not fully recapitulate chronic environmental exposure via drinking water. This route was selected to ensure precise and reproducible systemic dosing, avoiding the high inter-individual variability in gastrointestinal absorption and palatability-driven intake fluctuations inherent to oral administration. While this approach strengthens mechanistic interpretation of the ERS-autophagy-apoptosis axis by minimizing pharmacokinetic noise, future studies incorporating oral or dietary exposure models are warranted to validate the translational relevance of our findings to real-world environmental scenarios.

## 5 Conclusion

This study confirms that Cd exposure induces liver injury in rats and activates autophagy through the PERK, IRE1α, and ATF6 signaling pathways. FAM134B is a key ER autophagy receptor. It mediates selective recognition and clearance of damaged ER fragments during this process. ERS is not only an important signal in Cd-induced liver injury but also exerts cellular self-defense by regulating FAM134B-dependent reticulophagy. In turn, autophagy alleviates ERS through feedback regulation. This forms a bidirectional protective loop between ERS and autophagy. The loop jointly participates in the defense against hepatocellular damage under Cd exposure.

## Data Availability

The data presented in this study are available on reasonable request from the corresponding author.
